# Nestin Modulates Glucocorticoid Receptor Function by Cytoplasmic Anchoring

**DOI:** 10.1371/journal.pone.0006084

**Published:** 2009-06-29

**Authors:** Rudolph Reimer, Heike Helmbold, Beata Szalay, Christian Hagel, Heinrich Hohenberg, Wolfgang Deppert, Wolfgang Bohn

**Affiliations:** 1 Heinrich-Pette-Institute for Experimental Virology and Immunology at the University of Hamburg, Hamburg, Germany; 2 Institute of Neuropathology, Universitätsklinikum Hamburg-Eppendorf, Hamburg, Germany; Dresden University of Technology, Germany

## Abstract

Nestin is the characteristic intermediate filament (IF) protein of rapidly proliferating progenitor cells and regenerating tissue. Nestin copolymerizes with class III IF-proteins, mostly vimentin, into heteromeric filaments. Its expression is downregulated with differentiation. Here we show that a strong nestin expression in mouse embryo tissue coincides with a strong accumulation of the glucocorticoid receptor (GR), a key regulator of growth and differentiation in embryonic development. Microscopic studies on cultured cells show an association of GR with IFs composed of vimentin and nestin. Cells lacking nestin, but expressing vimentin, or cells expressing vimentin, but lacking nestin accumulate GR in the nucleus. Completing these networks with an exogenous nestin, respectively an exogenous vimentin restores cytoplasmic anchoring of GR to the IF system. Thus, heteromeric filaments provide the basis for anchoring of GR. The reaction pattern with phospho-GR specific antibodies and the presence of the chaperone HSC70 suggest that specifically the unliganded receptor is anchored to the IF system. Ligand addition releases GR from IFs and shifts the receptor into the nucleus. Suppression of nestin by specific shRNA abolishes anchoring of GR, induces its accumulation in the nucleus and provokes an irreversible G1/S cell cycle arrest. Suppression of GR prior to that of nestin prevents entry into the arrest. The data give evidence that nestin/vimentin specific anchoring modulates growth suppression by GR. We hypothesize that expression of nestin is a major determinant in suppression of anti-proliferative activity of GR in undifferentiated tissue and facilitates activation of this growth control in a precise tissue and differentiation dependent manner.

## Introduction

The class IV IF-protein nestin [1] is a well known marker of embryonic stem cell derived progenitors that have the potential to develop into neuroectodermal, endodermal and mesodermal lineages [2].

In the developing brain nestin is found coexpressed with vimentin from neural tube closure until the end of gliogenesis. Nestin is not only present in early developmental stages, it reappears transiently in adult tissue after injury to muscle [3], brain [4], [5] and liver [6], situations requiring high proliferative activity of undifferentiated cells to repair the damaged tissue. Thus, nestin is also a major marker of regenerating tissue.

The close relationship between nestin expression and proliferative activity in tissue suggests that functions of nestin exceed those of increasing the mechanical stability of the cell. Indeed, phosphorylation and structural organization of nestin are highly dynamic throughout the cell cycle [7]. Nestin promotes phosphorylation-dependent disassembly of vimentin IFs during mitosis [8], and it interacts with Cdk5/p35 [9], a kinase regulating differentiation of neuronal and muscle cells. Downregulation of nestin in neuronal cells activates Cdk5/p35 dependent apoptosis, suggesting that nestin is a survival determinant, protecting neuronal progenitors from stress induced cell death [10].

In intial studies on the distribution of nestin in mouse embryo tissue we detected a coincident strong accumulation of GR in nestin positive cells. Therefore, we asked whether this may indicate a structural or even functional relationship. GR is the cellular mediator of hormone regulated stress in the body and a key regulator of growth and differentiation in embryonic tissue, notably the brain, the immune system, bone, cartilage, and muscle [11], [12], [13], [14]. Some studies emphasized a role of microtubules in intracellular transport of activated GR into the nucleus [15], [16]. Purified tubulin showed direct binding of receptor molecules [17], [18], [19]. FKBP 52, a chaperone which associates with GR, has an affinity for dynein, a microtubule dependent cytoplasmic motor protein [20], [21]. Thus, there is evidence that microtubules control intracellular trafficking of the hormone activated receptor. But, neither disruption of microtubules nor of microfilaments was sufficient to induce nuclear localization of the unliganded receptor, nor did it impair nuclear transport of the liganded receptor [22], [23]. Thus, microtubules are involved in nuclear transport of GR, but not necessarily control retention of GR in the cytoplasm. Particulate receptor heterocomplexes isolated from the cytosol of mouse L-cells also contained the IF protein vimentin in addition to actin and tubulin [24], [25].

Here we show, that intermediate filaments composed of nestin and vimentin mediate cytoplasmic anchoring specifically of the unliganded receptor in undifferentiated cells. Anchoring of GR to these filaments is of functional relevance as suppression of nestin is sufficient to activate an irreversible growth arrest, a function which is overcome by concomitant suppression of GR. The finding is consistent with the hypothesis that nestin acts as a survival factor, impeding negative regulation of growth by GR in undifferentiated cells.

## Results

### Nestin colocalizes with GR in embryonic mouse tissue

**Figure 1:**
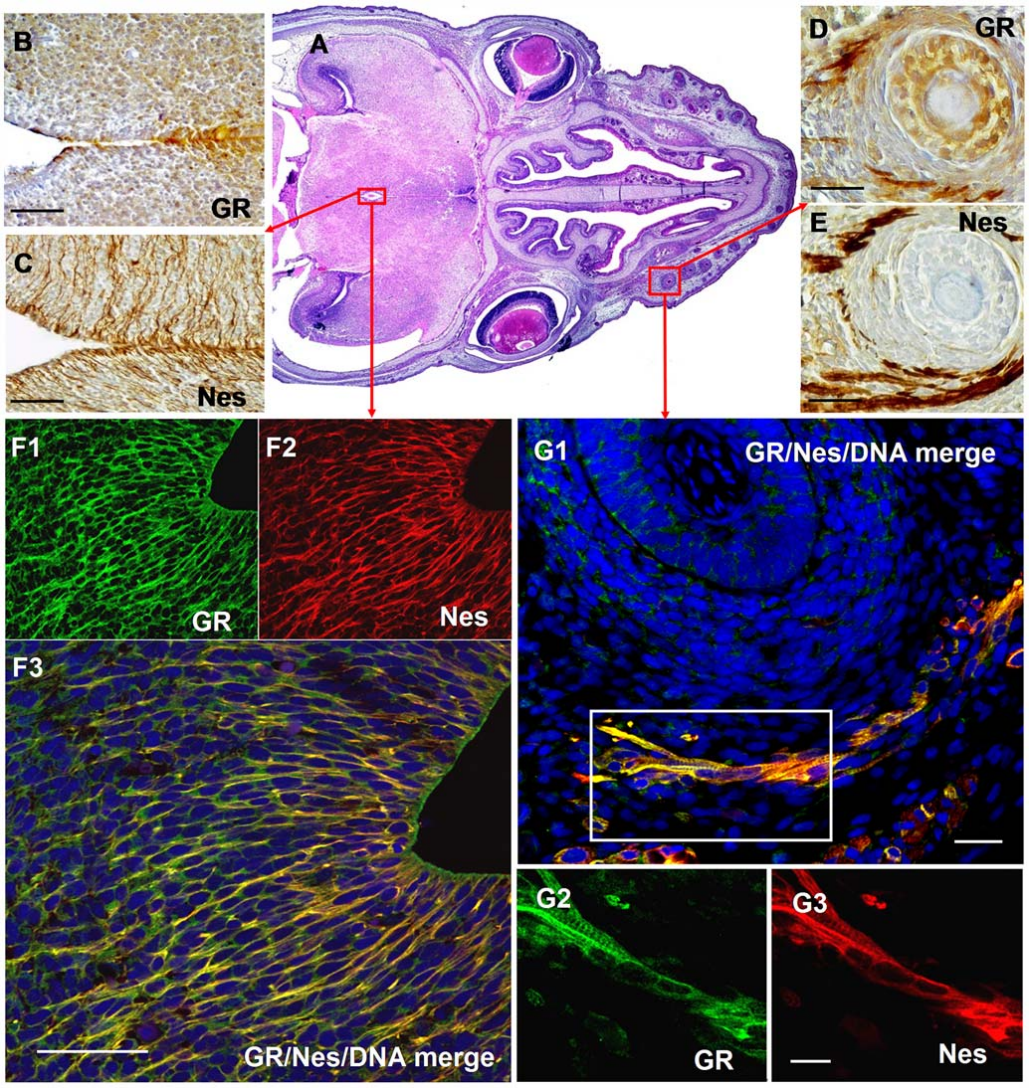
Nestin and GR colocalize in embryonic tissue of mice. (A) Horizontal section through the head of a mouse embryo (E 14.5); paraffin section stained with hematoxilin-eosin; (B–E) Immunoperoxidase labelling of GR and Nes on consecutive sections demonstrates expression in glia (B and C) and muscle fibres at vibrissae (D and E). (F–G) Fluorescence double labelling demonstrates co-localization of GR and Nes in glia (F1–F3) and in developing muscles adjacent to a hair follicle (G1–G3). Bars in A–E = 100 µm; G1 = 20 µm; G3 = 10 µm.

Immunoperoxidase labelling of nestin or GR on consecutive sections of mouse embryo heads (E14.5) demonstrated strong nestin and GR signals in neural cells lining the third ventricle (Figure 1, A, B, and C) and in muscle cells adjacent to a hair follicle (Figure 1, A, D, and E). Confocal microscopy revealed a strong nestin signal (red color) in the neuroepithelium and in radial glia (Figure 1F). This is in accordance with previous findings of others showing selective presence of nestin in areas of continuous neurogenesis, namely the lateral wall of the lateral ventricle and in ependymal and subventricular cell layers [26], [27], [1]. Nestin staining clearly coincided with the staining of GR (green color). In addition, a coincident staining of both proteins was found in muscle cells adjacent to a hair follicle (Figure 1G). The data suggest that nestin positive cells in developing tissue accumulate significant amounts of the glucocorticoid receptor.

### Nestin and GR costain in undifferentiated human blood cells

**Figure 2:**
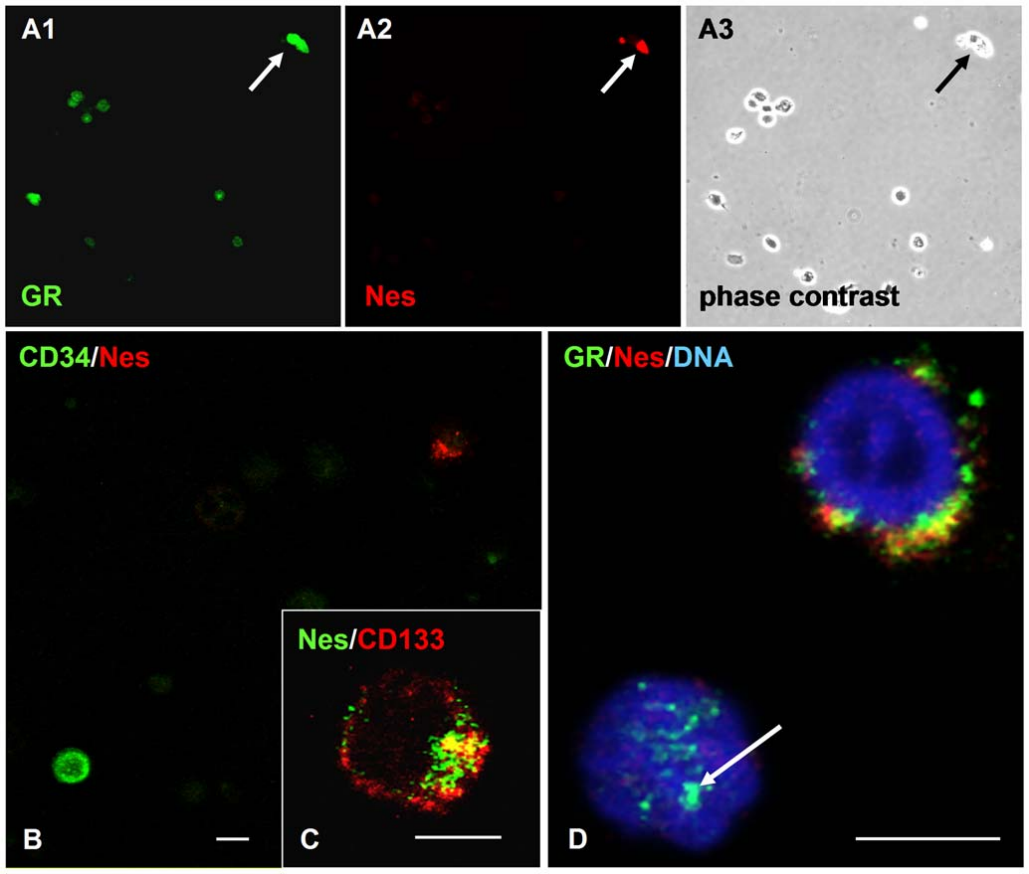
Human PBMCs anchoring GR express nestin and the stem cell marker CD133. (A–D) Confocal images of PBMCs isolated from cord blood; (A1–A3) cells were attached to glass coverslips, fixed with acetone, and double labelled with antibodies to GR and Nes. The proportion of Nes positive cells is less than 1%. (B) Nes positive cells are negative for the hematopoietic lineage marker CD34.(C) Nes positive cells express the stem cell marker CD133 (merged image). (D) Merged image of cells extracted with a non-ionic detergent, fixed with paraformaldehyde, double labelled with antibodies to GR and Nes and incubated with the DNA stain DRAQ5 [79]. Cytoplasmic GR in Nes expressing cells resists extraction with a non-ionic detergent, whereas Nes negative cells retain GR only in the nucleus (arrow). Bars in B, C, and D = 5 µm.

Individual steps in blood cell differentiation are well defined by specific markers, making it an ideal system to determine the relationship between nestin/GR colocalization and the status of cell differentiation. Nestin expression is known to identify blood precursor or stem cells [28]. Here we assayed peripheral blood mononuclear cells (PBMCs) isolated from human cord blood for this phenotype. CD34 and CD133 were used as markers of early stages of blood cell differentiation. Only a small proportion (less than 1%) of human cord blood cells was nestin positive (Figure 2, A1–A3). Nestin positive cord blood cells were negative for CD34, an early marker of the hematopoietic lineage and *vice versa* (Figure 2B), but they expressed the stem cell marker CD133 (Figure 2C). When cord blood cells were extracted with a nonionic detergent, only those cells which were positive for nestin and CD133 retained GR in the cytoplasm (Figure 2D). Cells which lacked nestin showed a nuclear GR signal (Figure 2D, arrow). The data indicate that cytoplasmic retention of GR in nestin expressing PBMCs defines adult stem or progenitor cells.

### Nestin dependent retention of GR in mouse embryo fibroblasts is a marker of early passages

**Figure 3:**
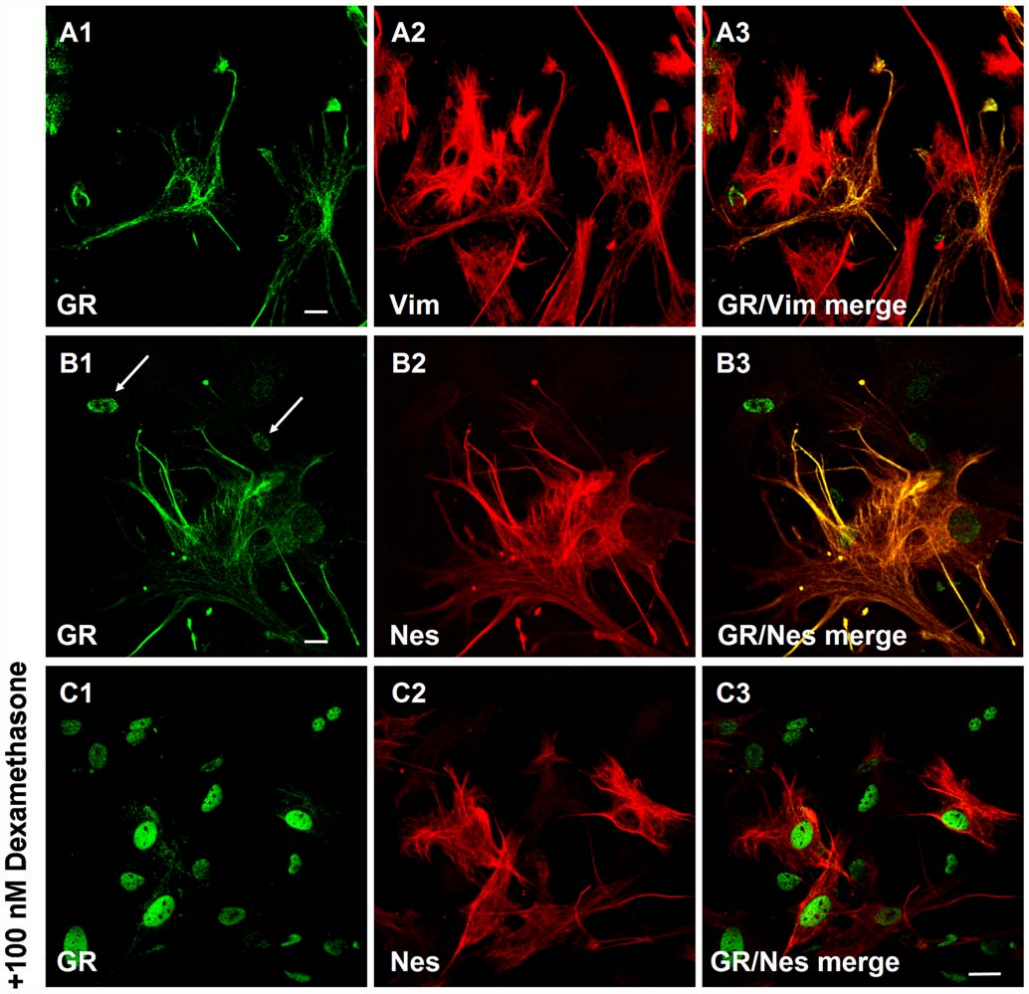
GR colocalizes with nestin and vimentin in embryonic mouse fibroblasts. Fibroblasts were explanted from Balb/C embryos (E 14.5). (A1–A3) Coincident labelling of cytoplasmic GR and Vim is confined to a subpopulation of cells. (B1–B3) Cytoplasmic accumulation of GR identifies Nes expressing cells. (C1–C3) Incubation with the GR ligand dexamethasone (100 nM) abolishes co-staining of GR with Nes to the benefit of a nuclear GR staining. Bar in Fig. 3, A–C = 5 µm.

We next asked if the coincident presence of nestin and GR is a stable phenotypic marker that is maintained after explantation of primary cells in tissue culture. Mouse embryo fibroblasts [29] were isolated from BALB/c embryos (E 14.5). At the 2nd passage after explantation about 5 to 20% of the cells in a culture showed a coincident staining of nestin and GR, and both colocalized with the vimentin filament system. (Figure 3, A and B). In nestin negative cells the receptor localized to the nucleus (Figure 3, B1, arrows). Addition of the GR ligand dexamethasone abolished the GR signal on the nestin/vimentin filament system and induced an accumulation of GR in the nucleus (Figure 3C). The proportion of nestin expressing cells declined gradually during passaging, decreasing to less than 1% nestin positive cells at the 6th passage, whereas vimentin expression was maintained. Concomitant with downregulation of nestin, the cells accumulated GR in the nucleus, adopted a senescent phenotype characterized by a flattened cell shape, positive staining for senescence associated β-galactosidase and entry into a sustained growth arrest (data not shown).

Similarily, primary cell cultures derived from the hippocampus of embryonic rats (E 14.5 ), a proliferative region of the embryonic brain with significant nestin-immunoreactivity [11], [30] contained cells which expressed nestin in addition to vimentin and showed a cytoplasmic accumulation of GR (data not shown). Nestin negative cells exhibited a nuclear GR signal only.

The data indicate that cytoplasmic retention of GR in embryonic cells coincides with expression of nestin and vimentin and is a feature of early passages. It is lost concomitantly with adoption of a senescent phenotype.

### Anchoring of GR to IFs requires the coincident presence of vimentin and nestin

**Figure 4:**
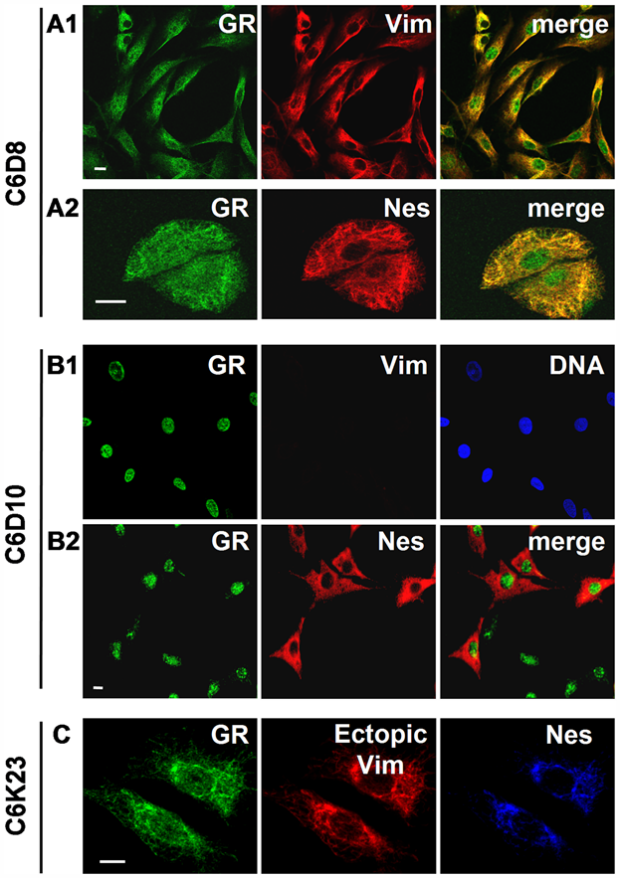
Retention of GR on the cytoskeleton requires vimentin and nestin. (A1–A2) The Vim expressing subclone C6D8 accumulates GR in the cytoplasm. Cytoplasmic GR colocalizes with Nes. (B1) GR in the Vim deficient subclone C6D10 is only nuclear. (B2) A proportion of the Vim negative C6D10 cells expresses Nes. (C) Expression of an exogenous mouse Vim protein in Vim deficient cells restores cytoplasmic accumulation of GR and colocalization of GR, Nes, and Vim. Bars in A–C = 5 µm.

**Figure 5:**
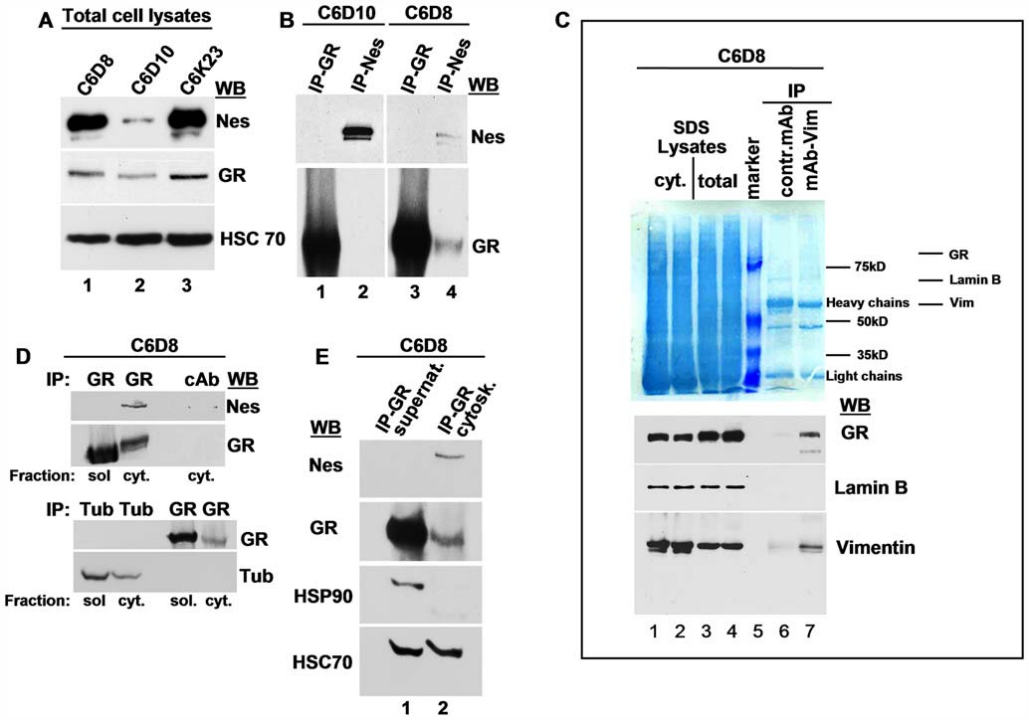
GR from the cytoskeleton fraction of anchoring competent cells coimmunoprecipitates with nestin, vimentin, and HSC70. (A) SDS lysates of cells. Anchoring competent cells (C6D8 and C6K23) contain significant amounts of nestin. (B) Significant amounts of nestin are immunoprecipitated from the detergent soluble fraction of vimentin deficient C6D10 cells (B, lane 2) but not of vimentin positive C6D8 cells (B, lane 4); nestin solubilized from C6D8 cells coimmunoprecipitates with GR (B, lane 4). (C) Immunoprecipitation (IP) with the monoclonal vimentin antibody V9 shows co-precipitation of GR (C, lane 7); nuclei are excluded from the immunoprecipitate as indicated by the absence of lamin B; a monoclonal antibody to measles virus protein was used for control IP(C, lane 6); Figure 5C, upper part, shows the nitrocellulose sheet after protein transfer and staining with Amido black; the lower part shows the same membrane after incubation with goat anti-vimentin, rabbit anti-lamin B, and rabbit anti-GR antibodies and detection of bound antibodies with enzyme conjugated antibodies; (cyt) cytoskeleton fraction, (total) whole cell lysate; the relative position of the labelled proteins on the Amido black stained sheet is marked. (D) Western blot analysis (WB) indicates the presence of Nes in GR immunoprecipitates derived from the cytoskeleton fraction, but shows no coimmunoprecipitation of GR and tubulin (Tub). (E) GR derived from the cytoskeleton fraction coimmunoprecipitates with HSC70 (E, lane 2); GR precipitated from the supernatant lysis buffer coprecipitates also with HSP90 (E, lane 1).

To determine the need of nestin and vimentin for GR anchoring we analyzed cell lines varying in IF-protein composition. Rat C6 subclones which either express vimentin or completely lack cytoplasmic IFs [31], [32] were used to determine this relationship in detail. GR was present in the cytoplasm of C6D8 cells, a subclone which contains an IF network composed of vimentin and nestin (Figure 4, A1). In contrast, GR was strictly nuclear in C6D10 cells, which lack cytoplasmic IFs (Figure 4, B1). About 40% of the cells in a C6D10 culture displayed a diffuse nestin staining in the cytoplasm (Figure 4, B2). C6D10 cells contained significantly lower amounts of nestin than vimentin positive cells (Figure 5A). To determine an association of GR with the cytoskeleton the cells were lysed *in situ* by use of the detergent Triton-X100. GR staining was retained on cytoskeletons of extracted C6D8 cells (Figure 4, A2). Confocal microscopy and colocalization analysis pointed to coincident staining patterns of GR and vimentin in these cells (Figure S1, A1–A3). In contrast, neither tubulin (Figure S1, B1–B2) nor actin (Figure S1, C1–C2) colocalized with GR, as indicated by the broad scatter diagrams. We subjected C6D8 cells to treatment with cytochalasin B, a drug which disrupts the actin filament system, but leaves the vimentin filament system intact. In these cells the GR label still colocalized with vimentin filaments and did not redistribute with actin into patches (Figure S2).

To substantiate the role of the IF-system in GR anchoring we restored the vimentin filament network in C6D10 cells by stably expressing an exogenous mouse vimentin protein. The exogenous protein contained an epitope in the rod domain which facilitated its detection by the human vimentin specific monoclonal antibody VIM3B4 [33]. Endogenous rat vimentin protein was identified by reaction with the monoclonal antibody V9, which binds to rat but not to mouse vimentin [33]. Cells (C6K23) with the reconstituted vimentin filament system showed a filamentous co-staining of GR, vimentin and nestin (Figure 4C). In addition, vimentin and GR could be co-immunoprecipitated from the cytoskeleton fraction of C6D8 cells (Figure 5C, lane 7). The precipitate was negative for lamin B, indicating that the presence of GR did not result from co-immunoprecipitation of residual nuclei. Tubulin and GR could not be co-immunoprecipitated (Figure 5D), which is consistent with the confocal data, showing no colocalization of GR and tubulin (Figure S1, B1–B2). Thus, the data substantiate a role of vimentin in cytoplasmic anchoring of GR.

Subsequently, we asked whether anchoring of GR to the IF system could be modulated in dependence of nestin. Western blots of total cell lysates revealed a strong nestin signal in the anchoring competent C6D8 and C6K23 cells (Figure 5A, lanes 1 and 3), whereas only a weak signal was present in total lysates of the IF deficient C6D10 cells (Figure 5A, lane 2). Immunoprecipitation experiments done with the detergent soluble fraction of C6D10 cells showed no co-precipitation of GR and nestin, despite the fact that significant amounts of nestin were precipitated (Figure 5B, lanes 1 and 2). In contrast, only low amounts of nestin were immunoprecipitated from lysates of C6D8 cells, and these complexes contained GR (Figure 5B, lane 4). The data indicate that nestin in the IF deficient C6D10 cells is soluble and not associated with GR. In contrast, nestin in the vimentin positive C6D8 cells is insoluble, and the minor amounts which could be released with the mild extraction conditions were associated with GR.

**Figure 6:**
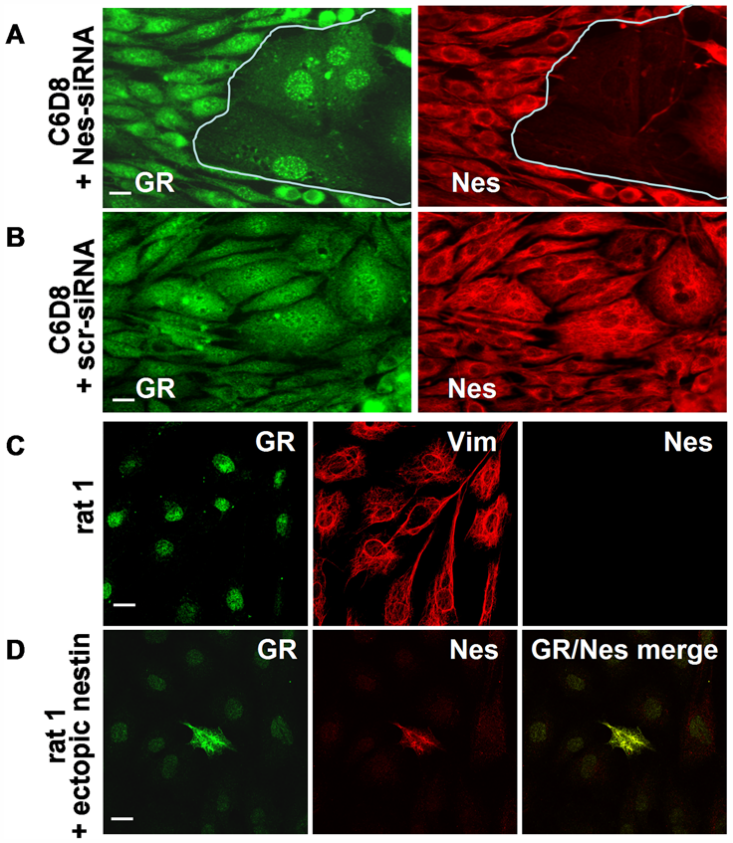
Cytoplasmic localization of GR depends on the presence of nestin. (A) Suppression of Nes expression in C6D8 cells by transfection with nestin specific siRNA abolishes cytoplasmic GR staining; only nuclear GR staining is left. (C) GR is only nuclear in rat 1 cells which contain vimentin, but lack nestin. (D) Ectopic expression of nestin in rat1 cells induces cytoplasmic accumulation of GR. Bars in A, B, C, F, and G = 5 µm.

To substantiate the relationship between presence of nestin and vimentin, and retention of GR in the cytoplasm we depleted nestin from C6D8 cells by use of specific siRNA. As shown in Figure 6A, siRNA transfected cultures showed areas where cells were nestin negative (marked by a white line). GR became exclusively nuclear in these nestin depleted cells and the cells flattened and enlarged. No such changes were obtained by transfection with a scrambled siRNA (Figure 6B).

In another approach we expressed an exogenous nestin protein in rat1 cells. Rat 1 cells contain a vimentin filament system but completely lack nestin and show GR exclusively in the nucleus (Figure 6C). When rat1 cells were transfected with a nestin expression vector, GR accumulated in the cytoplasm (Figure 6D). The data indicate that co-expression of nestin and vimentin is required to provide a structural basis for cytoplasmic anchoring of GR.

**Figure 7:**
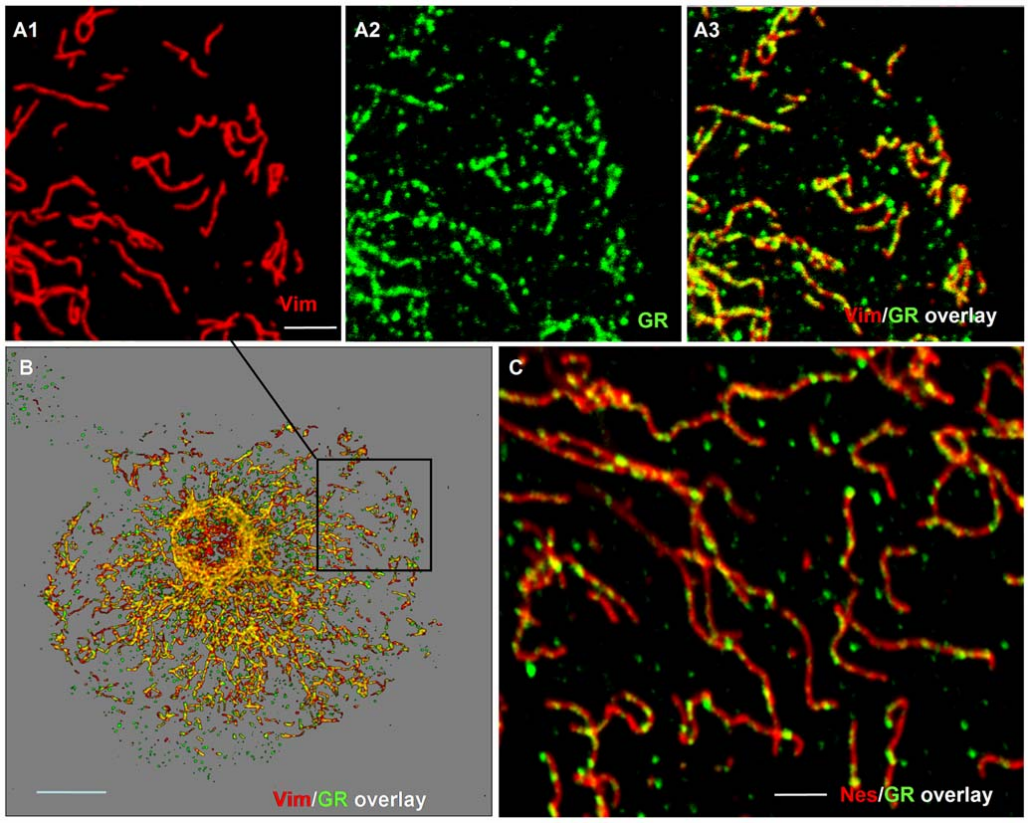
GR redistributes with shortened IFs composed of a tailless vimentin protein. (A–D) Confocal images of cytoskeletons prepared from B8 VimMT3B4 cells, which stably express a tailless vimentin protein; tailless vimentin forms a fragmented IF network (A, B); the filaments contain also nestin (C); GR colocalizes with vimentin/nestin filament fragments (A, C); Bars in A and C = 1 µm; B = 5 µm.

Further to substantiate the association of GR with IFs we asked whether altering the structure of the vimentin filament network would induce a coincident rearrangement of GR. We transfected the vimentin deficient C6D10 cells with an expression vector coding for a C-terminal truncated vimentin protein (ct-vimentin). Although not essential for the polymerization itself the tails seem to stabilize the lateral interaction of vimentin filament subunits. In addition, they seem to mediate the structural interaction of vimentin with other major cytoskeleton filament systems [34]. As shown in Figure 7A–C tailless vimentin was still capable of forming a filament network, but this was composed of shortened filaments and less well organized. GR colocalized with these shortened vimentin filaments. High resolution confocal images showed that the GR label did not evenly decorate the vimentin filaments but exhibited a spotty distribution (Figure 7, A3). A similar staining pattern was obtained in double labeling of nestin and GR (Figure 7C).

Quantitative colocalization analysis of confocal microscopy data revealed a high degree of colocalization between GR and tailless vimentin, respectively GR and nestin. We performed both, an intensity correlation coefficient-based analysis and an object based approach. The statistical significance test based on 200 image randomizations [35] showed a significant true colocalization with a P = 100% probability for GR and tailless vimentin respectively GR and nestin. The calculated Pearson's correlation coefficients were r = 0,825 for GR and tailless vimentin, respectively r = 0,757 for GR and nestin. The distance based analysis [36] revealed 89% positive thresholded pixels in the GR channel and 73% positive thresholded pixels in the vimentin channel. The calculated results for GR/Nes-colocalization were 91% positive thresholded pixels in the GR channel and 84% positive thresholded pixels in the Nestin channel. The data verified that GR was not randomly distributed but colocalized with the filaments composed of tailless vimentin and nestin.

### Localization of IF associated GR complexes at the EM level

**Figure 8:**
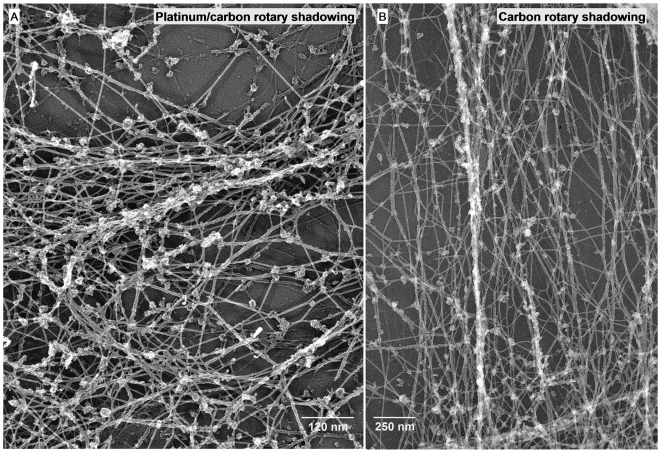
Visualization of the cytoskeleton architecture by EM replica technique. C6D8 cells were grown on glass coverslips, extracted with a non-ionic detergent, fixed with aldehyde, critical point dried, rotary shadowed with platinum/carbon (A) respectively carbon (B), detached from the glass, and transferred onto EM grids. The micrographs reveal maintenance of the 3D organization of the networks with this technique.

**Figure 9:**
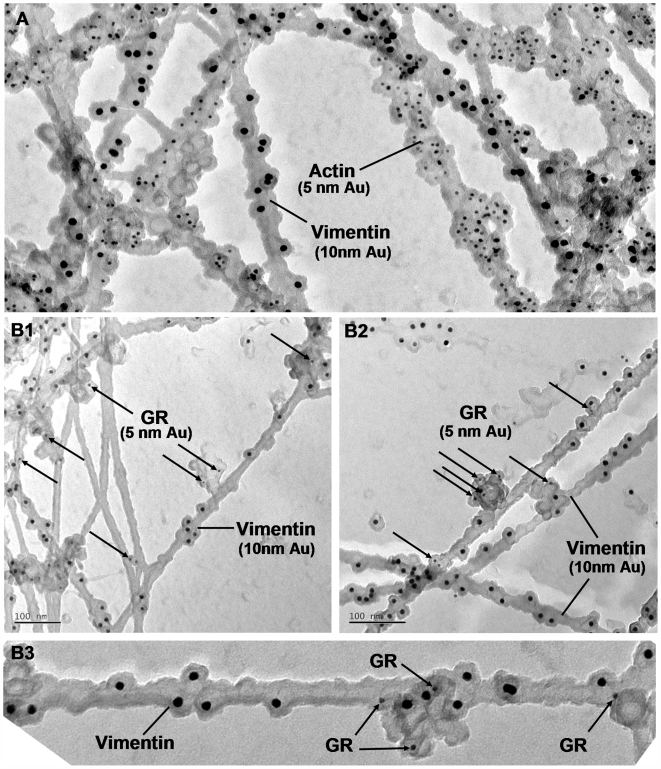
EM immunogold replica technique shows specific association of GR containing complexes with vimentin filaments. Cytoskeletons of C6D8 cells were fixed, double labelled with antibodies and immunogold particles, postfixed, and handled the same way as described in legend to Figure 8. (A) The labeling technique clearly identifies vimentin containing IFs (10 nm immunogold particles); labeling of actin with 5 nm immunogold particles; (B1–B3) double labelling with antibodies to vimentin (10 nm immunogold particles) and GR (5 nm immunogold particles) shows specific association of GR with vimentin containing filaments.

To proof unequivocally that GR is bound to individual IF filaments we assayed its localization at an ultrastructural level by electron microscopy. We did an immunogold labelling analysis on cultured C6D8 cells by replica EM. The cells were extracted with detergent under controlled conditions, fixed, labelled with antibodies, critical point dried and shadowed with platinum carbon or only carbon[37], [38]. The technique fills the gap between low resolution light microscopy and high resolution electron microscopy and is highly suited to understand changes in cytoskeleton architecture in relation to dynamic processes in the cell [39]. As shown in Figure 8 the procedure well preserved the 3D organization of the filament network. Immunogold labelling combined with carbon shadowing enabled us to distinguish vimentin filaments (10 nm immunogold particles) from other filament types, such as actin filaments (5 nm immunogold particles) (Figure 9A). Double labelling of GR (5 nm immunogold particles) and vimentin (10 nm immunogold particles) revealed an association of the GR label specifically with the vimentin containing filaments (Figure 9, B1–B3). The results suggest that GR is part of a larger complex bound to distinct sites at the vimentin/nestin filaments.

### The non-activated receptor form is bound to the cytoskeleton

To define the functional status of the IF associated GR we asked whether nestin/vimentin associated GR complexes colocalize with HSC70 and HSP90, the major chaperone molecules stabilizing the conformation of GR in the activation cycle. Confocal microscopy indicated colocalization of GR and HSC70 (Figure S3), whereas HSP90 could not be detected on cytoskeletons. Accordingly, only HSC70 was present in GR immunoprecipitates obtained from the cytoskeleton fraction (Figure 5E, lane 2). In contrast, GR which was immunoprecipitated from the detergent soluble fraction contained both, HSC70 and HSP90 (Figure 5E, lane 1).

Association with HSC70 indicates that the filament bound receptor is inactive, but at an advanced stage in the activation cycle [40], [41]. To further confirm this suggestion we assayed its reaction with certain antibodies that are specific for the nonactivated, respectively the activated form.

**Figure 10:**
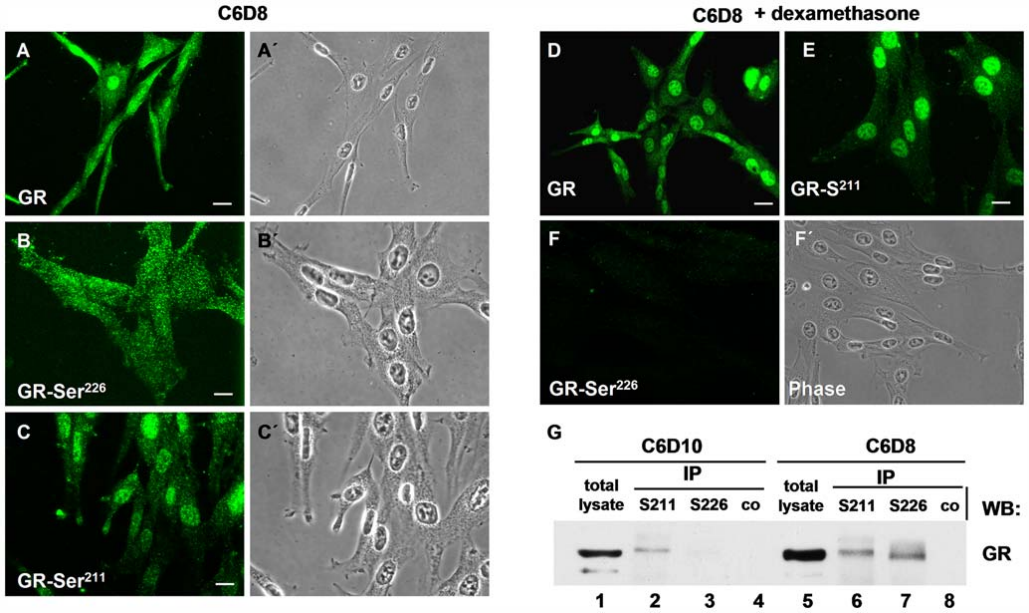
The non-activated GR is retained on the cytoskeleton. (A) A non-phospho specific GR antibody detects nuclear and cytoplasmic GR in Triton X-100 extracted cells. (B) GR-Ser^226^ specific antibodies recognize cytoskeleton bound GR in extracted cells. (C) GR-^Ser211^ specific GR antibodies primarily recognize nuclear bound GR. (D–F) Incubation of C6D8 cells with a GR specific ligand (dexamethasone) abolishes cytoplasmic staining with the GR-^Ser226^ antibody (F) and strengthens the nuclear staining with GR-^Ser211^ specific GR antibodies (E). (G) Immunoprecipitation (IP) of GR with phosphospecific antibodies indicates reaction with GR subfractions, differing in electrophoretic mobility. GR precipitated from anchoring deficient C6D10 cells primarily reacts with the GR-^Ser211^ antibody. Bars in A–F = 5 µm.

GR is phosphorylated at three major sites on its N-terminus, namely Ser^203^, Ser^211^, and Ser^226^ in human GR, corresponding to Ser^224^, Ser^232^, and Ser^246^ in rat GR [42], [43]. Phosphorylation of Ser^211^ is required for full transcriptional activity and correlates with a strong phospho-Ser^211^ staining in the nucleus, suggesting that Ser^211^ is a marker for activated GR *in vivo* [43], [44]. In contrast, phosphorylation of Ser^226^ correlates with an increased receptor nuclear export in the absence of hormone. In addition, it decreases the transcriptional GR activity in response to a hormone, suggesting that it holds GR in the non-activated state [45], [46]. Thus, we asked whether we could distinguish cytoskeleton bound and nuclear GR by reaction with these antibodies. A non-phospho specific GR antibody reacted with cytoplasmic as well as with nuclear GR in extracted C6D8 cells (Figure 10A). In contrast, the phospho-Ser^226^ antibody only detected cytoplasmic GR (Figure 10B), whereas the phospho-Ser^211^ antibody showed a dominant nuclear reaction in these cells (Figure 10C). In C6D8 cells treated with dexamethasone prior to Triton X-100 extraction, only a strong nuclear phospho-Ser^211^staining was present (Figure 10E), whereas phospho-Ser^226^ staining was lacking (Figure 10F). In accordance, GR could be immunoprecipitated with the phospho-Ser^226^ antibody only from lysates of C6D8 but not of C6D10 cells (Figure 10G). In contrast, the phospho-Ser^211^ antibody precipitated GR from lysates of both, CD10 and C6D8 cells (Figure 10G). The data indicate that the non-activated form of GR identified by reaction with the phospho-Ser^226^ antibody is retained on the cytoskeleton.

### Suppression of nestin induces nuclear localization of GR and a sustained growth arrest

**Figure 11:**
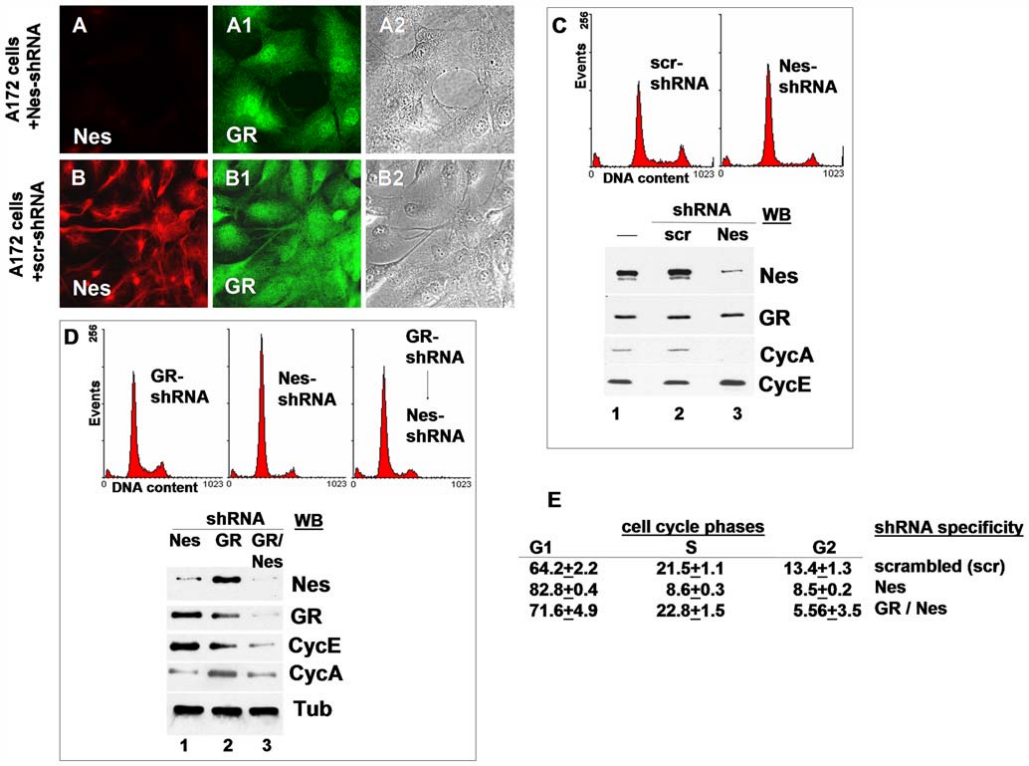
Suppression of nestin by RNA interference induces a G1/S cell cycle arrest. (A, A1, A2) Human A172 cells transduced with nestin specific shRNA (Nes-shRNA) show loss of nestin expression (A) and increased nuclear GR staining (A1); (B, B1; B2) localization of Nes (B) and GR (B1) in A172 cells transduced with scrambled shRNA (scr-shRNA). (C) Cells expressing Nes-shRNA are arrested in G1/S transition, indicated by the altered cell cycle profile, the decrease in cyclin A expression, and maintenance of cyclin E expression (C, lane 3). (D) Suppression of GR in advance impairs activation of a G1/S arrest by Nes-shRNA. Cyclin A is detectable, cyclin E does not accumulate to high levels (D, lane 3). (E) Proportions of cell cycle phases in A172 cultures expressing scrambled, Nes specific, or in a sequential manner GR and Nes specific shRNA. Mean values and SDs are of three independent experiments, counting 10,000 cells in each experiment; bar in A and B = 10 µm.

In late passages of MEFs, when the cells had ceased proliferation and adopted a senescence like phenotype, nestin positive cells were hardly detectable. This let us ask whether loss of nestin could be causally related to activation of growth suppression. To evaluate this hypothesis we assayed the impact of nestin loss on cell growth in A172 cultures, a human cell line expressing nestin and vimentin and showing cytoplasmic anchoring of GR to this filament system. Nestin was suppressed by transduction of the cells with nestin specific shRNA (compare Figure 11, A and B). Suppression was obvious in Western-blot already 24 h post transduction (data not shown). Concomitantly, in immunofluorescence the nuclear GR signal increased relative to the cytoplasmic signal (compare Figure 11, A1 and B1). Flow cytometry analysis showed an increase in the proportion of G1 cells in cultures transduced with nestin shRNA, whereas the proportion of S-phase and G2 phase cells declined (Figure 11, C and E). The cyclin pattern revealed a decrease in the cyclin A level in nestin shRNA transduced cells, whereas the cyclin E level was maintained or even increased (Figure 11C, lane 3). Nestin shRNA transduced cells could not be subcultivated in contrast to cells transduced with a scrambled (scr) shRNA. It indicates that suppression of nestin arrested A172 cells irreversibly in G1/S.

To determine if growth suppression induced by loss of nestin required GR, we depleted cells of GR by use of shRNA, and subsequently transduced these cells with nestin specific shRNA. Although GR and nestin were suppressed (Figure 11D, lane 3), cyclin A was still detectable, cyclin E stayed at a lower level than in cells transduced with nestin shRNA alone, and a significant proportion of cells were in S-phase (Figure 11E).

This indicates that cells depleted of GR were less efficiently arrested in G1/S upon suppression of nestin, compared to treatment with nestin shRNA alone. The data substantiate the hypothesis that anchoring of GR by nestin compromises its growth suppressive functions.

## Discussion

Our data indicate that nestin in conjunction with vimentin determines cytoplasmic accumulation of the glucocorticoid receptor, a major sensor of stress response at the cellular level. Neither nestin nor vimentin were sufficient on its own, but anchoring required their copolymerization into an IF system. Nestin, due to its short N-terminal domain, cannot self-assemble into filaments, but forms heteromeric filaments with other class III IF-proteins, such as vimentin, desmin, or GFAP, with nestin prosumably locating at the filament periphery [47], [48], [49], [50], [51]. Therefore, a certain structural configuration of the IFs resulting from copolymerization of nestin and vimentin seems to be required for an efficient binding of a GR containing complex. Our data at least indicate that the tail domain of vimentin is not essential for anchoring of GR.

All of the cell types we have tested so far for cytoplasmic anchoring of GR expressed the class III IF protein vimentin. In differentiation, vimentin expression often precedes that of the tissue specific IF protein, suggesting that vimentin could have a specific role in conjunction with nestin. Further studies must show whether other class III IF proteins can substitute for vimentin in this function.

The differential reaction of phosphospecific GR antibodies gives evidence that specifically the non-activated GR form is anchored to the vimentin/nestin filament system. This form is in a hypophosphorylated state, as deduced from its increased mobility in SDS-PAGE. Any reaction with this phosphor site specific antibody was abolished in the presence of dexamethasone which shifted GR into the nucleus. The data indicate that GR anchored to the IF system is not irreversibly withdrawn from the activation cycle but awaits binding of a ligand.

It is well known that the activation status of the receptor in the cytoplasm is also defined by its association with chaperone molecules. Binding of chaperone molecules is necessary for the receptor to gain the high affinity, steroid binding form. GR-chaperone complexes are highly unstable and undergo constant cycles of dissociation and association. As we show here, GR containing immunecomplexes obtained with nestin antibodies from the cytoskeleton fraction of anchoring competent cells contained the chaperone HSC70, but not HSP90. The immunoprecipitation assay was limited by the low solubility of nestin/vimentin IFs under the mild conditions, applied to generate fractions for IP. However, HSP90 was also not observed on the cytoskeleton by immunocytochemical staining *in situ* staining, whereas HSC70 coincided with GR present on the cytoskeleton. In contrast nestin was isolated at significant quantities by immunoprecipitation from vimentin deficient cells. But this sample showed no coprecipitation of GR. GR complexes isolated from the detergent soluble fraction contained HSP90 in addition to HSC70.

According to the current model, which largely results from in vitro studies with purified proteins, the presence of HSC70 in a GR containing complex indicates that GR associated with the IF system is in a certain advanced stage in the activation cycle. HSC70 and the subsequently bound HSP90 induce the formation of a deep hydrophobic cleft in the ligand binding domain of GR, required for hormone binding [52], [53], [54]. After ligand binding the chaperone molecules are displaced from the receptor molecules, the receptor dimerizes and translocates into the nucleus [55], [56], [57].

The data raise the question whether chaperone molecules themselves may mediate cytoplasmic retention. But previous studies of others showed that GR and chaperone molecules can move together into the nucleus suggesting that association with chaperones is not sufficient for cytoplasmic retention [58], [21]. In addition, overexpression of the glucocorticoid receptor raised the nuclear level of naïve, chaperone associated receptor molecules [59], [60]. These data contradict a direct role of chaperone molecules in cytoplasmic retention of GR.

Our data give evidence that anchoring of GR to nestin/vimentin is related to the maintenance of a high proliferation rate. A first, indirect hint was obtained with mouse embryo fibroblasts. Anchoring of GR in these primary cells was typical of early passages, when the proliferation rate was high. In advanced passages the cells ceased expression of nestin, contained GR in the nucleus, and concomitantly halted proliferation. It suggested a link between nestin expression, anchoring of GR and maintenance of proliferative capacity. Our RNAi experiments corroborate the suggestion. First, suppression of nestin abolished cytoplasmic localization of GR and induced a sustained growth arrest. Secondly, the growth suppressive effect could be rescued by blocking GR expression prior to that of nestin. The data extend a recent report, showing that suppression of nestin in repopulating mesangial cells lowered the number of BrdU incorporating cells [61]. Thus nestin/vimentin dependent anchoring can modulate growth suppression by GR.

The coincident accumulation of nestin and GR in embryonic mouse tissue let us assume that nestin/vimentin dependent anchoring is of relevance for tissue development. It could be a tool to provide high amounts of receptor molecules by downregulating nestin expression and to activate GR mediated growth suppression during differentiation in a precise time and tissue specific manner. GR is known to play a critical role in regulating proliferative activity during embryonic development. Ligand activated GR was reported to impair proliferation and differentiation of neuronal progenitor cells *in vivo* and *in vitro* [11], [62], [63], to induce a G1 cell cycle arrest or apoptosis in immature thymocytes [64], and to impair proliferation of fibroblasts [65] and undifferentiated mammary epithelial cells [66], [46]. Accumulation of GR in undifferentiated cells reaches the highest level just prior to terminal differentiation [67], [68], [69]. The GR knock-out mouse shows several defects and dies at birth from respiratory dysfunction at a time when brain development is not complete. The lungs display increased cell proliferation rates, suggesting that GR is essential to regulate proliferation during tissue development [70]. The outcome of a conditional GR knock-out in the developing mouse mamma gave evidence that GR loss does not globally affect proliferation of the targeted cell type, but does so at a certain stage in development and differentiation [71]. Regarding the function of glucocorticoids in the developing brain, the interpretation of in vivo data is complicated by the fact that basal levels of glucocorticoids are essential for neuronal development, plasticity and survival, whereas stress levels of glucocorticoids produce neuronal loss [72].

Our data obtained with populations of naive human blood cells suggest that nestin/vimentin dependent anchoring of GR appears very early in the differentiation pathway. Anchoring was typical of cells which were CD133 positive, but CD34 negative. The CD133^+^/CD 34^−^ phenotype characterizes very primitive peripheral cells, that are capable of generating differentiated cells of the neuroectoderm, as well as liver, lung, brain, heart, gut, and striated muscle cells [28], [73]. These blood cells have *in vitro* mesenchymal potential and a high hematopoietic activity [74].

In summary, we hypothesize that cytoplasmic anchoring of GR to nestin/vimentin filaments endows undifferentiated cells with significant amounts of unliganded GR that are used to activate GR functions in dependence of the developmental stage. Regulatory elements in the *nestin* promoter, which specify its tissue specific expression [75], [76] could be of primary importance in this control mechanism.

## Materials and Methods

### Cell culture

C6D10 and C6D8 cells were subcloned from the C6 rat glioma cell line (ATCC (CLL107) [32]. C6K23 cells express an exogenous mouse vimentin protein that contains the Vim3B4-epitope [33]. B8 VimMT3B4 cells stably express an exogenous mouse vimentin protein lacking the vimentin tail domain (introduction of a stop codon by base exchange at position 1358/9 of mouse vimentin cDNA). Rat 1 cells and rat 2 cells were kindly provided by W. Ostertag, Heinrich-Pette-Institute. The cells were grown with Dulbecco's Modified Eagle's Medium (DMEM), supplemented with 5% fetal calf serum (FCS). Primary mouse cells prepared from Balb/C mouse embryos (E14.5) were kindly provided by Christina Heinlein, Heinrich-Pette-Institut. Hippocampal explants of rat embryos (E14.5) were kindly provided by M. Schweitzer, Center for Molecular Neurobiology, Hamburg, Germany; the explants were prepared as described by Neidhardt et al. [77]. Human peripheral blood mononuclear cells (PBMC) and human cord blood cells were isolated by Ficoll-Hypaque density centrifugation. Isolated cells were washed twice in RPMI 1640 and resuspended to give 1×10^6^ cells/ml in RPMI 1640 containing 10% FCS. PBMCs were incubated overnight at 37°C to separate adherent cells. Non adherent cells were collected and washed once with RPMI 1640 supplemented with 10% FCS.

### Antibodies, expression plasmids and transfections

The following antibodies were used: mouse monoclonal anti-vimentin V9 and Vim3B4 (Boehringer Mannheim, Germany); goat anti-vimentin and rabbit anti-lamin B (P. Traub, Ladenburg, Germany); anti-glucocorticoid receptor M20 (sc-1004), E20 (sc-1003) and H300 (sc-8992) (Santa Cruz Biotechnology, Santa Cruz, U.S.A.); anti-nestin R-20 (sc-21249) and C-20 (sc-21247) (Santa Cruz Biotechnology); mouse anti-tubulin (Oncogene Research, San Diego, U.S.A.), mouse anti-CD34 (BD Biosciences, Belgium), (mouse anti-CD133 (Mitenyi Biotec, Germany). Normal goat serum and normal donkey serum were purchased from DAKO (Glostrup, Danmark); donkey anti-mouse IgG, donkey anti-rabbit IgG, and donkey anti-goat antibodies conjugated with Alexa 488, 568, 555, or 633 (Molecular Probes, Leiden, Netherlands) were used as secondary antibodies. DRAQ5™ (Biostatus Limited, Leicestershire, England) was used to stain DNA. The expression vector for tailless vimentin was kindly provided by Peter Traub, Ladenburg, Germany. The nestin expression plasmid was kindly provided by J. Yang [78].

### Immunocytochemistry

Cells grown on glass coverslips were extracted *in situ* with pre-warmed 80 mM Pipes buffer pH 6.8 [1 mM MgCl_2_, 4 mM EGTA, 4% polyethylene glycol 6000 (PEG 6000), 1% Triton X-100, 1% Trasyslol (Bayer, Leverkusen, Germany), 10 µg/ml Leupeptin). Extracted cells were washed shortly with this buffer lacking the detergent and fixed with 1% paraformaldehyde in this buffer for 15 min.

Intact cells were fixed in cold (−20°C) acetone for 30 min. An alternative approach was to fix the cells with 1% paraformaldehyde in phosphate buffered saline (PBS) for 20 min and to permeabilize subsequently with 0.2% Triton X-100 in this buffer. Residual free aldehyde groups were blocked with 50 mM glycine in PBS for 10 min. Acetone fixed cells were rehydrated in PBS for 10 min, blocked with 2% normal serum for 20 min, and labeled with the primary antibody. Immunogold labelling of cytoskeletons and handling of the probes for electron microscopy were done as described in detail elsewhere [37]. Goat anti-mouse- and goat anti-rabbit gold conjugates were purchased from British Biocell International, Cardiff, UK.

### Flow cytometry

Cells grown on 100 mm Petri dishes were trypsinized, washed once with PBS, resuspended in 0.5 ml PBS and fixed in 70% cold methanol. After rehydration in PBS, the cells were suspended in 1 ml of propidium iodide solution (5 µg propidium iodide, 1 mg RNase A per ml of PBS) for 30 min at 37°C. Labeled cells were run through a Coulter Epics XL-MCL Counter (Beckman-Coulter, U.S.A.). The raw data were converted into single-parameter histograms using the WinMDI 2.8 software and saved as FCS files. The quantitative cell cycle analysis was performed with the Cylchred program (University of Cardiff, England).

### Image acquisition and image processing

Images were taken with the confocal microscope Zeiss LSM 510 Meta (Zeiss, Jena, Germany). Image processing was done with Photoshop version 7.01, and with ImageJ 1.33 (NIH, public domain, U.S.A.). Image processing included the following steps:(i) correction of tonal value including adjustment of threshold and gamma value, (ii) noise suppression with low pass filters, (iii) sharpening through overlay of highpass (Laplace) and bandpass filtered original images. Merged images were generated by setting signals below the threshold value to 50% gray. Quantitative colocalization analysis, including the determination of Pearson's correlation coefficient were done with raw confocal data. Image data were acquired at Nyquist sampling rates. Iterative deconvolution was done, when necessary, with the Zeiss Advanced Imaging Microscopy (AIM) Software v. 3.2 in an automated modus. Colocalization analysis and scattergramms were calculated with the Zeiss Advanced Imaging Microscopy (AIM) Software v. 3.2 and JACoP [36] under ImageJ v. 1.41o.

### Immunoprecipitation and Western blotting

Subconfluent cultures were washed twice with cold (4°C) PBS. The cells were scraped from the culture dish and lysed with 1 ml of Hepes-buffer pH 7.0 (150 mM NaCl; 1 mM EDTA; 1 mM EGTA; 0.1% NP40; 1% aprotinin ( Bayer; Leverkusen, Germany); 10 µg/ml Leupeptin) while shaking on ice for 30 min. The lysates were cleared in an Eppendorf microfuge at 13,000 rpm for 30 min. The protein concentration in the supernatant lysate was determined colorimetrically with the ‘BCA Protein Assay’ reagent (Pierce, Rockford, USA). An aliquot containing 100 µg of protein was adjusted to a total volume of 300 µl with lysis buffer. The probes were incubated for 2 h at 4°C with 2 µg of the appropriate antibody prebound to 100 µl of a 10% slurry of protein G-sepharose. The PGS with the bound immune complexes was washed three times with cold lysis buffer. Precipitated proteins were dissolved in 20 µl SDS sample buffer, electrophoresed on 8% respectively 10% polyacrylamide gels, and transferred onto nitrocellulose (Hybond, Amersham, England). The nitrocellulose sheets were stained with Amido black, destained, and blocked with 5% low fat milk in Tris-buffered salt solution (20 mM Tris/HCl, pH 7.6; 150 mM NaCl) containing 0.05% Tween-20. Proteins were detected with the appropriate primary antibody and a secondary, enzyme conjugated antibody, using the ECL Western-blot detection kit (Amersham, Braunschweig, Germany). Chemiluminescence was visualized by exposing the membrane to X-ray films.

### Silencing of GR and nestin

The lentiviral control vector DNA containing a scrambled sequence and the vector DNA for silencing human GR and human nestin were obtained from Sigma, Germany. Lentiviral particles were produced in 293T cells by cotransfection of 3 µg vector DNA, 3 µg packaging construct pCMV-dR8.91 [obtained from D. Trono, University of Geneva], and 0.5 µg pMD2a expression construct. Viral particles were harvested after 48 h and stored at −80°C. For silencing of GR, THP-1 cells were infected with 500 ng (p24) lentiviral vector and selected for stably transduced cells with puromycin.

## Supporting Information

Figure S1 GR colocalizes with vimentin in cytoskeletons of C6D8 cells. (A–C) Confocal images of cytoskeletons labelled with antibodies to vimentin (Vim), glucocorticoid receptor (GR), actin and tubulin (Tub); intensities below threshold value were set to 50% gray. Colocalization analysis was performed with raw confocal data. (A1–A3) Double staining with antibodies to GR and vimentin; (B1–B2) triple staining with antibodies to GR, actin and vimentin; (C1–C3) triple staining with antibodies to GR, tubulin, and vimentin; scatter diagrams indicate coincident signals of GR and vimentin (inserts in A3, B1, and C2), but not of GR and actin (insert in B1) or GR and tubulin (insert in C1). Bar in B = 5 µm; bars in A1and C1 = 1 µm.
(5.50 MB TIF)

Figure S2 GR remains associated with the vimentin network in cells treated with cytochalasin B. Confocal images of cells; cells were incubated with 5 µg/ml cytochalasin B for 20 min, lysed in situ with Triton X-100 and stained with antibodies to vimentin, actin and GR; bar = 10 µm.
(2.59 MB TIF)

Figure S3 Cytoskeleton bound GR colocalizes with HSC70. Confocal image of an extracted cell double labelled with antibodies to GR and HSC70. Bar = 1 µm.
(2.37 MB TIF)
