# Supplementary figures and images for: Nestin Modulates Glucocorticoid Receptor Function by Cytoplasmic Anchoring
Source: PLoS One. 2009 Jun 29;4(6):e6084. doi: 10.1371/journal.pone.0006084 (PMC2698154; doi:10.1371/journal.pone.0006084)

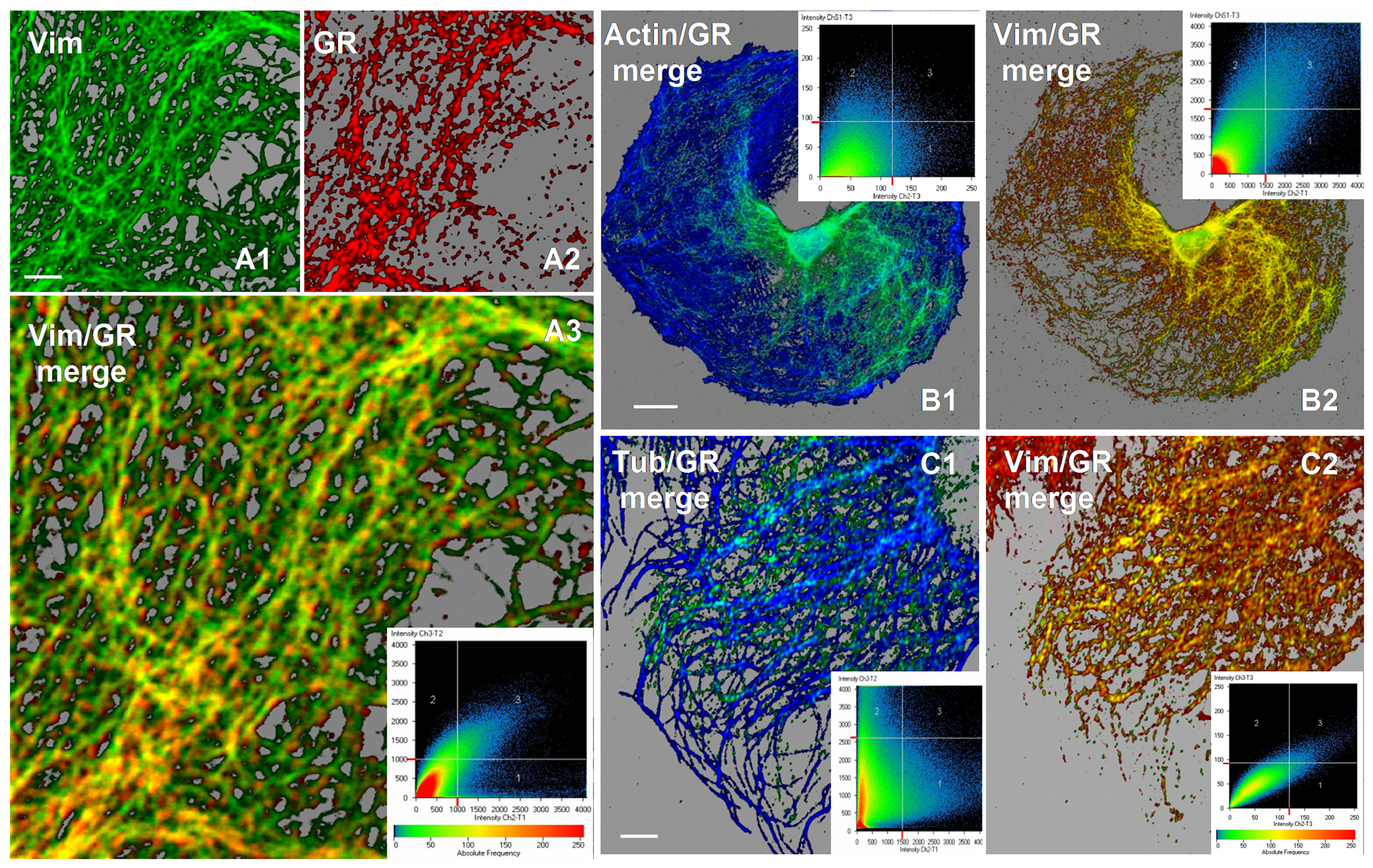

Supplement: Figure S1 — GR colocalizes with vimentin in cytoskeletons of C6D8 cells. (A–C) Confocal images of cytoskeletons labelled with antibodies to vimentin (Vim), glucocorticoid receptor (GR), actin and tubulin (Tub); intensities below threshold value were set to 50% gray. Colocalization analysis was performed with raw confocal data. (A1–A3) Double staining with antibodies to GR and vimentin; (B1–B2) triple staining with antibodies to GR, actin and vimentin; (C1–C3) triple staining with antibodies to GR, tubulin, and vimentin; scatter diagrams indicate coincident signals of GR and vimentin (inserts in A3, B1, and C2), but not of GR and actin (insert in B1) or GR and tubulin (insert in C1). Bar in B = 5 µm; bars in A1and C1 = 1 µm. (5.50 MB TIF) [file pone.0006084.s001.tif]

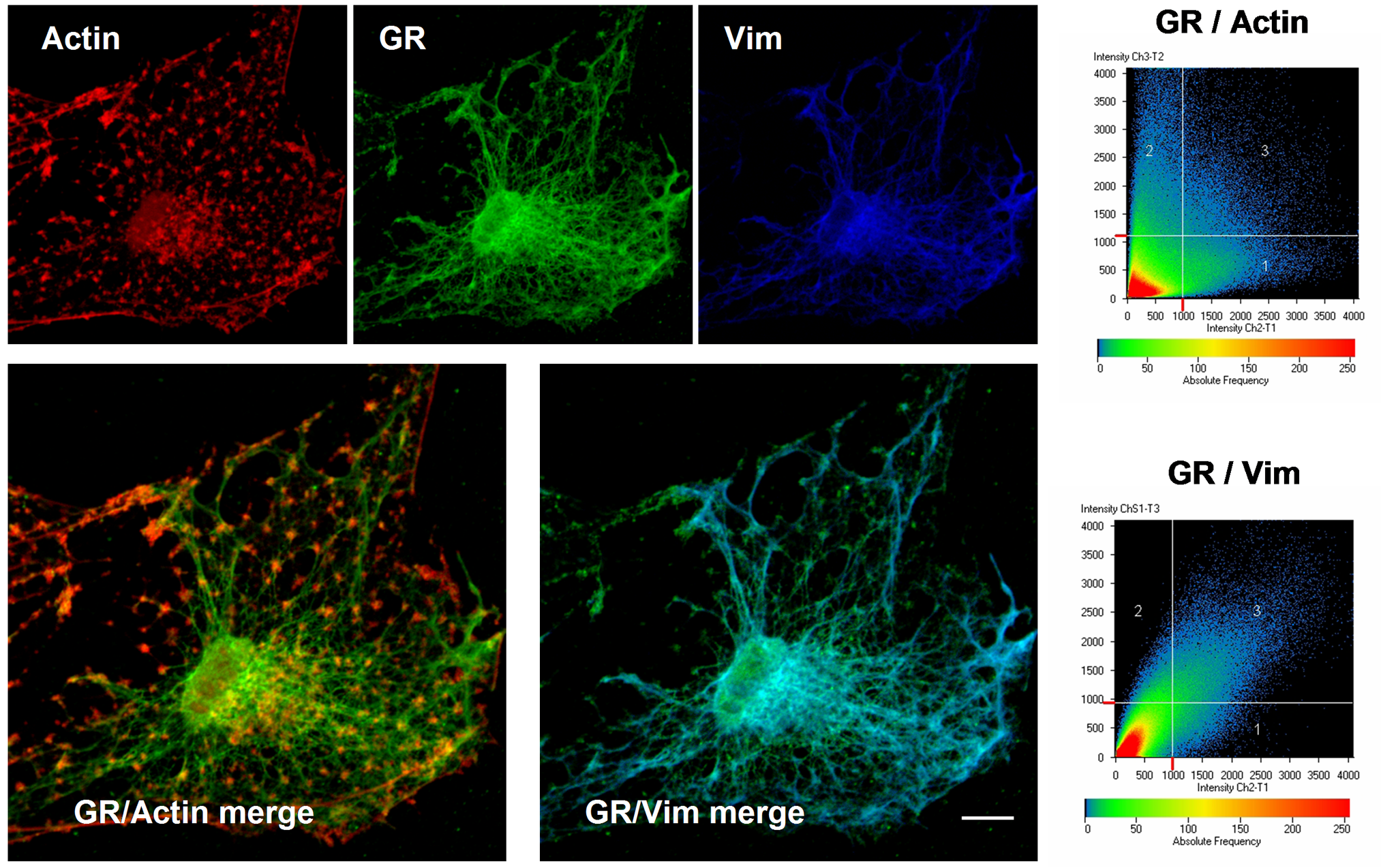

Supplement: Figure S2 — GR remains associated with the vimentin network in cells treated with cytochalasin B. Confocal images of cells; cells were incubated with 5 µg/ml cytochalasin B for 20 min, lysed in situ with Triton X-100 and stained with antibodies to vimentin, actin and GR; bar = 10 µm. (2.59 MB TIF) [file pone.0006084.s002.tif]

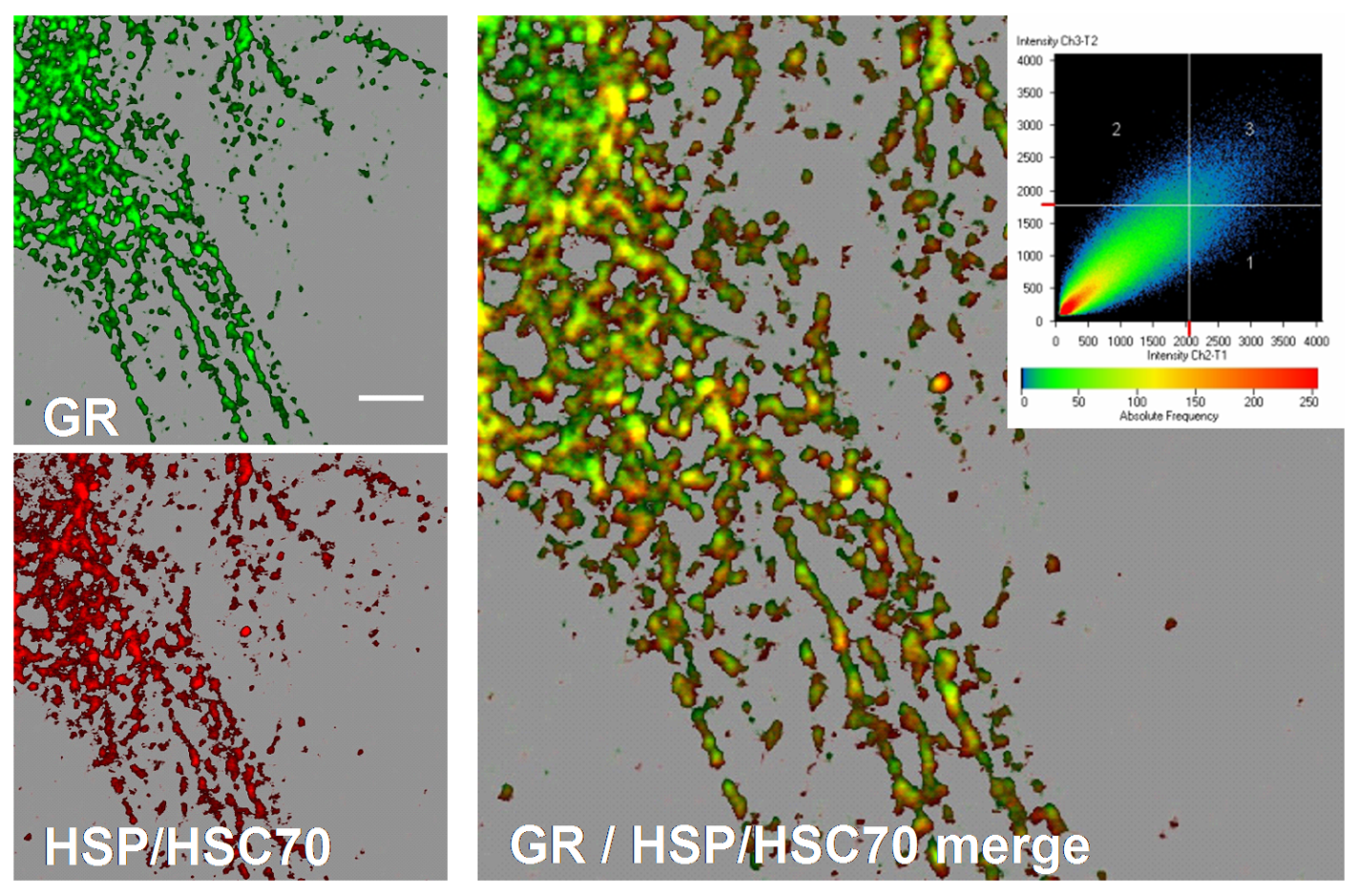

Supplement: Figure S3 — Cytoskeleton bound GR colocalizes with HSC70. Confocal image of an extracted cell double labelled with antibodies to GR and HSC70. Bar = 1 µm. (2.37 MB TIF) [file pone.0006084.s003.tif]
